# Brain Network Prediction of Outcomes for Family Caregiver Depression

**DOI:** 10.1093/geroni/igaf122.3890

**Published:** 2025-12-31

**Authors:** Belise Swartwood, Felipe Jain, Benjamin Wade

**Affiliations:** The General Hospital Corporation, Boston, Massachusetts, United States; Massachusetts General Hospital / Harvard Medical School, Boston, Massachusetts, United States; Massachusetts General Physicians Organization, Inc., Boston, Massachusetts, United States

## Abstract

Alzheimer’s disease and related dementias affect over seven million Americans. Family members who provide care and assistance to their relatives often experience psychological burden and emotional distress, including elevated rates of depression. Caregiver response to depression treatment is highly variable, resulting in delays and inefficacious interventions for many, but there are no validated biomarkers to guide therapy. Overall brain function and depression treatment response is thought to be determined in part by the strength of large-scale brain connectivity networks. Resting-state functional magnetic resonance imaging (fMRI) can estimate these connectivity strengths. In a pilot study, we examined whether baseline connectivity could predict depressive symptom improvement following Mentalizing Imagery Therapy (MIT) or control conditions in family caregivers of people living with dementia (N = 43). Participants underwent resting-state fMRI and completed a depression measure before and after treatment. Independent Component Analysis extracted 45 functional connectivity components per participant. Least Absolute Shrinkage and Selection Operator (LASSO) regression with 10-fold cross-validation identified features associated with changes in QIDS scores. SHAP values were obtained to determine feature strength. Permutation testing (1,000 iterations) assessed statistical significance. Connectivity patterns within several networks—including the dorsal and ventral default mode networks, a somatosensory network, and central executive network—were significantly associated with symptom change in combination with baseline depression and treatment group (R2=0.71; p < 0.05). These findings suggest that pre-treatment brain network connectivity may serve as a biomarker for identifying caregivers most likely to benefit from intervention. Such neurobiologically informed predictions might help personalize depression treatment for family caregivers.

